# Synthesis and crystal structure of *N*-(5-acetyl-4-methyl­pyrimidin-2-yl)benzene­sulfonamide

**DOI:** 10.1107/S2056989023001871

**Published:** 2023-03-15

**Authors:** Reham A. Mohamed-Ezzat, Benson M. Kariuki, Rasha A. Azzam

**Affiliations:** a Chemistry of Natural & Microbial Products Department, National Research Center, Cairo, Egypt; b School of Chemistry, Cardiff University, Main Building, Park Place, Cardiff CF10, 3AT, United Kingdom; c Department of Chemistry, Helwan University, Cairo, Egypt; Universität Greifswald, Germany

**Keywords:** synthesis, pyrimidine sulfonamide, crystal structure, X-ray diffraction

## Abstract

*N*-(5-Acetyl-4-methyl­pyrimidin-2-yl)benzene­sulfonamide, C_13_H_13_N_3_O_3_S, was sythesized and characterized by single-crystal X-ray diffraction. In the crystal, π–π inter­actions between the phenyl and pyrimidine groups of neighbouring mol­ecules form mol­ecular chains parallel to [010]. Adjacent mol­ecular chains are linked by N—H⋯N hydrogen-bonding inter­actions between the pyrimidine and amine groups of neighbouring mol­ecules, resulting in a three-dimensional network.

## Abstract 2

*N*-(5-Acetyl-4-methyl­pyrimidin-2-yl)benzene­sulfonamide was synthesized and structurally characterized. In the crystal, π–π inter­actions between the phenyl and pyrimidine groups of neighbouring mol­ecules form mol­ecular chains parallel to [010]. Adjacent chains are linked by N—H⋯N hydrogen-bonding inter­actions, resulting in a three-dimensional network.

## 1. Chemical context

Sulfonamide-bearing mol­ecules with one or several pharmacological scaffolds constitute a class of drugs with anti­viral, anti­cancer, anti-carbonic anhydrase (CA), diuretic, cyclo­oxigenase 2 (COX2) inhibitory, protease inhibitory, and/or anti­bacterial activities (Supuran, 2003 ▸; Scozzafava *et al.*, 2003 ▸; Casini & Scozzafava, 2002 ▸). It is noteworthy that the sulfonamide moiety is one of the significant, privileged building blocks that medicinal chemists frequently find in potent drugs (Elgemeie *et al.*, 2019 ▸). Thus, many widely marketed drugs incorporate this moiety. Several pyrimidine sulfonamides and other pyrim­idine analogues that could be incorporated in new designs for bioactive mol­ecules with medicinal applications have already been considered (Azzam, 2019 ▸; Azzam & Elgemeie, 2019 ▸; Azzam *et al.*, 2017 ▸, 2019 ▸; Mohamed-Ezzat *et al.*, 2021 ▸, 2022 ▸; Elgemeie *et al.*, 2015*a* ▸,*b* ▸, 2017 ▸). The synthesis of *N*-(5-acetyl-4-methyl­pyrimidin-2-yl)benzene­sulfonamide (AMBS) was reported several decades ago (Gutsche *et al.*, 1964 ▸). In this article, we describe an alternative novel one-pot reaction methodology for the synthesis of this compound, which was also crystallized and crystallographically investigated. 
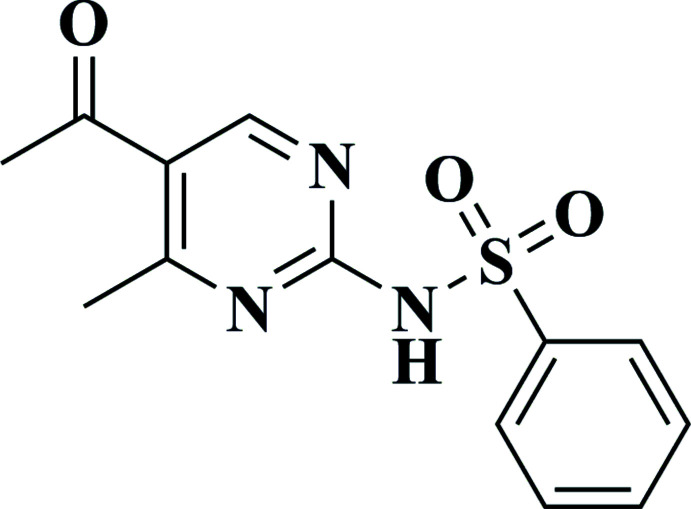


## 2. Structural commentary

**Figure 1:**
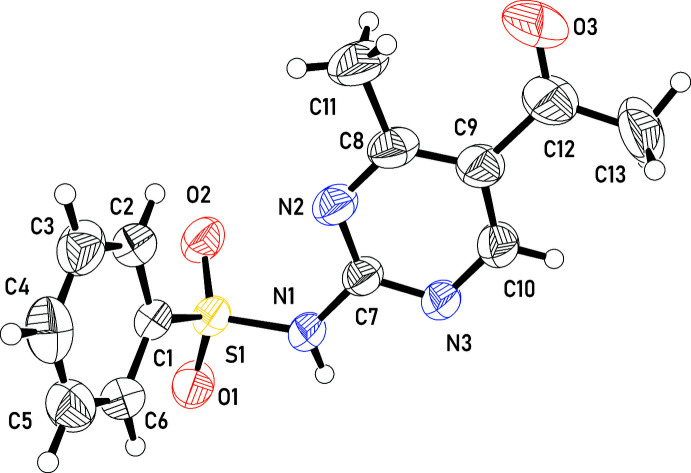
The mol­ecular structure of the title compound with atom labels and 50% probability atomic displacement ellipsoids.

*N*-(5-Acetyl-4-methyl­pyrimidin-2-yl)benzene­sulfonamide (AMBS) crystallizes in the monoclinic system, space group *P*2_1_/*c* and contains four mol­ecules in the unit cell (Z = 4). The asymmetric unit is shown in Fig. 1 ▸. The acetaldehyde group of the mol­ecule is disordered with two components related by a twist of 31.3 (1)° about the C_ar_—C bond. Apart from a slight twist of the aldehyde group associated with the disorder, the 1-(2-amino-4-methyl­pyrimidin-5-yl)ethan-1-one segment of the mol­ecule is essentially planar, the sulfonamide atom S1 being located only 0.423 (1) Å away from the plane of the pyrimidine group. The mol­ecule exhibits a C7—N1—S1—C1 torsion angle of −79.0 (2)°, while the twist between the planes of the phenyl group and the pyrimidine ring comprises a dihedral angle of 63.07 (7)°.

## 3. Supra­molecular features

**Figure 2:**
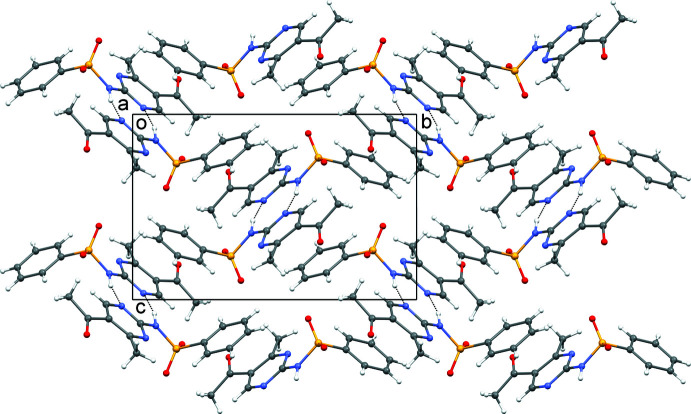
Crystal packing viewed down the *a* axis with N—H⋯N hydrogen bonds shown as dotted lines.

**Figure 3:**
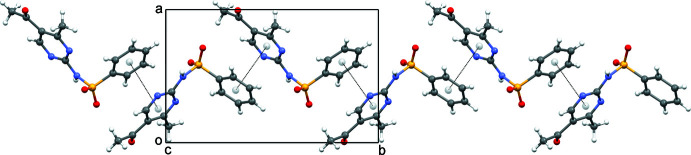
A segment of the crystal structure showing a chain of mol­ecules linked by π–π inter­actions. The dotted lines connect the centroids of the mol­ecules involved.

The packing of AMBS is shown in Fig. 2 ▸. In the crystal, partial π–π overlap is observed between the phenyl group of one mol­ecule and the pyrimidine group of an adjacent one related by 2_1_ symmetry (1 − *x*, − 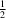 + *y*, 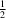 − *z* or 1 − *x*, 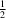 + *y*, 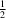 − *z*). The dihedral angle between the planes of the rings is 9.04 (10)° with a ring centroid-to-centroid distance of 3.769 (1) Å (Fig. 3 ▸). The slippage distances between the overlapping rings are 1.44 Å (1 − *x*, − 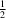 + *y*, 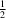 − *z*) and 1.58 Å (1 − *x*, 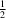 + *y*, 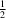 − *z*). These π–π inter­actions form chains in the structure in which one AMBS mol­ecule comprises the linker between two further mol­ecules. The bent nature of the mol­ecule results in a zigzag pattern of chains propagating parallel to [010].

The hydrogen-bonding inter­actions in the crystal are summarized in Table 1 ▸. Two linear N—H⋯N hydrogen bonds, with N⋯N distances of 2.891 (2) Å, occur between two neighbouring mol­ecules related by inversion symmetry (1 − *x*, 1 − *y*, 1 − *z*). A pair of hydrogen bonds is formed between the pyrimidine and amine groups of the two mol­ecules, resulting in a 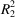 (8) geometry (Fig. 2 ▸). The hydrogen bonds link the mol­ecular chains formed by the π–π inter­actions and are perpendicular to the chains’ protrusion. Additionally, non-classical hydrogen-bonding contacts of the C—H⋯O type with C⋯O distances in the range of *ca* 2.7–3.4 Å help to consolidate the structure.

## 4. Database survey

A survey of the Cambridge Structural Database (Groom *et al.*, 2016 ▸; accessed February 2023) using *CONQUEST* (Bruno *et al.*, 2002 ▸) for structures containing the *N*-(pyrimidin-2-yl)benzene­sulfonamide group gave 164 hits, *i.e*. too many for them all to be analysed in detail.

An example of a closely related compound is 4,5,6-tri­meth­yl-2-[(phenyl­sulfon­yl)amino]­pyrimidine (TPAP) (ref­code VENKIJ; Li & Yang, 2006 ▸). In this structure, the dihedral angle between the planes through the phenyl and pyrimidine rings is 91.9°, larger than that observed for the title compound AMBS [63.07 (7)°]. In contrast to AMBS, π–π inter­actions are only observed between the pyrimidine rings in TPAP, resulting in stacking along the *a*-axis with inter­planar distances of 3.81 Å.

Another closely related compound is *N*-(pyrimidin-2-yl)benzene­sulfonamide (PBS) (refcode XIFKAZ01; Coles *et al.*, 2000 ▸). In PBS, the dihedral angle between the planes through the phenyl and pyrimidine rings is 74.5°, again larger than for AMBS. Also unlike in AMBS, π–π inter­actions occur in PBS between pairs of mol­ecules involving only the pyrim­idine rings and with an inter­planar distance of 3.5 Å. Similarly to AMBS, two linear N—H⋯N hydrogen bonds are observed in PBS between the pyrimidine and amine groups of neighbouring mol­ecules, resulting in similar 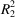 (8) motifs.

## 5. Synthesis and crystallization

**Figure 4:**
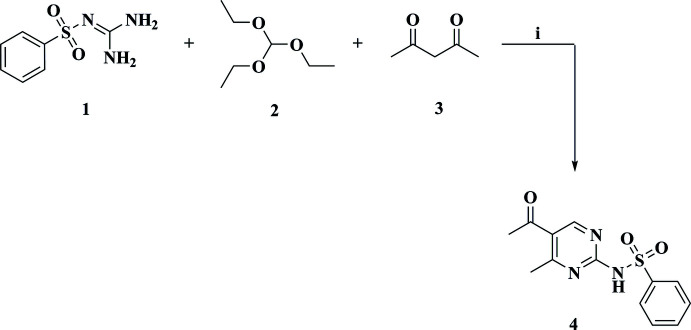
The synthesis of the title compound (**4**). Reagents & Conditions: (i) reflux; 6 h.

Phenyl­sulfonyl guanidine **1** is a common starting material for the synthesis of several heterocyclic compounds and has been utilized effectively in the generation of a range of biologically active compounds. Our approach was based on synthesizing the substituted sulfonyl derivative **4** by reacting the sulfonyl guanidine **1** with tri­ethyl­orthoformate **2** and acetyl acetone **3** (Fig. 4 ▸). The target product was identified by NMR spectroscopy and X-ray crystallography.

**Synthesis of compound 4:** Tri­ethyl­orthoformate (5 ml) was added to a mixture of phenyl­sulfonyl guanidine (0.05 mol) and acetyl acetone (0.1 mol). The reaction mixture was then refluxed for 6 h. After cooling, the resulting precipitate was filtered and crystallized from ethanol.

Orange crystals; yield 45%; m.p. 469 K. ^1^H NMR (400 MHz, DMSO-*d_6_*): δ 2.49 (*s*, 3H, CH_3_), 2.52 (*s*, 3H, CH_3_), 7.57–7.66 (*m*, 3H, Ar-H), 8.00–8.02 (*m*, 2H, Ar-H), 8.93 (*s*, 1H, CH-pyrimidine), 12.34 (*s*, 1H, NH). Analysis calculated for C_13_H_13_N_3_O_3_S (291.33): C, 53.60; H, 4.50; N, 14.42; S, 11.01. Found: C, 53.60; H, 4.49; N, 14.41; S, 11.00.

## 6. Refinement

Crystal data, data collection and structure refinement details are summarized in Table 2 ▸. The N—H hydrogen was refined freely. The remaining hydrogen atoms were positioned geometrically and using a riding model [C—H = 0.93–0.96 Å with *U*_iso_(H) = 1.2 or 1.5 *U*_eq_(C). The acetaldehyde group of the mol­ecule is disordered with two components related by a twist of 31.3 (1)° about the C_ar_—C bond. In the refinement, the two components were restrained to have similar geometry (SAME in *SHELXL*) and atomic displacement parameters (SIMU and ISOR). The occupancies of the two components refined to 0.591 (11)/0.409 (11).

## Supplementary Material

Crystal structure: contains datablock(s) I. DOI: 10.1107/S2056989023001871/yz2029sup1.cif

Structure factors: contains datablock(s) I. DOI: 10.1107/S2056989023001871/yz2029Isup3.hkl

Supporting information file. DOI: 10.1107/S2056989023001871/yz2029Isup3.cml

CCDC reference: 2245275

Additional supporting information:  crystallographic information; 3D view; checkCIF report

## Figures and Tables

**Table 1:** Hydrogen-bond geometry (Å, °)

*D*—H⋯*A*	*D*—H	H⋯*A*	*D*⋯*A*	*D*—H⋯*A*
N1—H1⋯N3^i^	0.83 (2)	2.06 (2)	2.891 (2)	179 (2)
C6—H6⋯O2^ii^	0.93	2.62	3.338 (3)	135
C10—H10⋯O1^i^	0.93	2.54	3.243 (3)	133
C11—H11*B*⋯O3*A*	0.96	2.26	2.769 (7)	113
C13*A*—H13*E*⋯O1^iii^	0.96	2.50	3.40 (3)	156

Symmetry codes: (i) 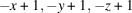 ; (ii) 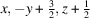 ; (iii) 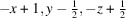 .

**Table 2:** Experimental details

Crystal data	
Chemical formula	C_13_H_13_N_3_O_3_S
*M*_r_	291.32
Crystal system, space group	Monoclinic, *P*2_1_/*c*
Temperature (K)	293
*a*, *b*, *c* (Å)	10.0699 (6), 14.7429 (6), 10.5212 (7)
β (°)	113.900 (7)
*V* (Å^3^)	1428.04 (16)
*Z*	4
Radiation type	Mo *K*α
μ (mm^−1^)	0.24
Crystal size (mm)	0.44 × 0.24 × 0.16

Data collection	
Diffractometer	Rigaku SuperNova, Dual, Cu at home/near, Atlas
Absorption correction	Gaussian (*CrysAlis PRO*; Rigaku OD, 2022 ▸)
*T*_min_, *T*_max_	0.484, 1.000
No. of measured, independent and observed [*I* > 2σ(*I*)] reflections	12642, 3532, 2548
*R*_int_	0.026
(sin θ/λ)_max_ (Å^−1^)	0.694

Refinement	
*R*[*F* ^2^ > 2σ(*F* ^2^)], *wR*(*F* ^2^), *S*	0.046, 0.125, 1.07
No. of reflections	3532
No. of parameters	216
No. of restraints	84
H-atom treatment	H atoms treated by a mixture of independent and constrained refinement
Δρ_max_, Δρ_min_ (e Å^−3^)	0.23, −0.29

Computer programs: *CrysAlis PRO* (Rigaku OD, 2022 ▸), *SHELXT* (Sheldrick, 2015*a* ▸), *SHELXL2018/1* (Sheldrick, 2015*b* ▸), *Mercury* (Macrae *et al.*, 2020 ▸) and *ORTEP-3 for Windows* (Farrugia, 2012 ▸).

## supplementary crystallographic information

### Crystal data

**Table:**

C_13_H_13_N_3_O_3_S	*F*(000) = 608
*M**_r_* = 291.32	*D*_x_ = 1.355 Mg m^−^^3^
Monoclinic, *P*2_1_/*c*	Mo *K*α radiation, λ = 0.71073 Å
*a* = 10.0699 (6) Å	Cell parameters from 5035 reflections
*b* = 14.7429 (6) Å	θ = 3.9–27.7°
*c* = 10.5212 (7) Å	µ = 0.24 mm^−^^1^
β = 113.900 (7)°	*T* = 293 K
*V* = 1428.04 (16) Å^3^	Block, orange
*Z* = 4	0.44 × 0.24 × 0.16 mm

### Data collection

**Table:**

Rigaku SuperNova, Dual, Cu at home/near, Atlas diffractometer	2548 reflections with *I* > 2σ(*I*)
ω scans	*R*_int_ = 0.026
Absorption correction: gaussian (CrysAlisPro; Rigaku OD, 2022)	θ_max_ = 29.5°, θ_min_ = 3.5°
*T*_min_ = 0.484, *T*_max_ = 1.000	*h* = −13→10
12642 measured reflections	*k* = −18→18
3532 independent reflections	*l* = −13→14

### Refinement

**Table:**

Refinement on *F*^2^	Primary atom site location: dual
Least-squares matrix: full	Hydrogen site location: mixed
*R*[*F*^2^ > 2σ(*F*^2^)] = 0.046	H atoms treated by a mixture of independent and constrained refinement
*wR*(*F*^2^) = 0.125	*w* = 1/[σ^2^(*F*_o_^2^) + (0.0493*P*)^2^ + 0.420*P*] where *P* = (*F*_o_^2^ + 2*F*_c_^2^)/3
*S* = 1.07	(Δ/σ)_max_ = 0.001
3532 reflections	Δρ_max_ = 0.23 e Å^−^^3^
216 parameters	Δρ_min_ = −0.29 e Å^−^^3^
84 restraints	

### Special details

**Table:**

Geometry. All esds (except the esd in the dihedral angle between two l.s. planes) are estimated using the full covariance matrix. The cell esds are taken into account individually in the estimation of esds in distances, angles and torsion angles; correlations between esds in cell parameters are only used when they are defined by crystal symmetry. An approximate (isotropic) treatment of cell esds is used for estimating esds involving l.s. planes.

### Fractional atomic coordinates and isotropic or equivalent isotropic displacement parameters (Å^2^)

**Table:**

	*x*	*y*	*z*	*U*_iso_*/*U*_eq_	Occ. (<1)
C1	0.5229 (2)	0.75192 (12)	0.26887 (19)	0.0461 (4)	
C2	0.6205 (2)	0.76291 (14)	0.2086 (2)	0.0552 (5)	
H2	0.630570	0.718727	0.150041	0.066*	
C3	0.7033 (2)	0.84155 (15)	0.2374 (3)	0.0651 (6)	
H3	0.768869	0.850929	0.196825	0.078*	
C4	0.6886 (3)	0.90550 (15)	0.3257 (3)	0.0712 (7)	
H4	0.746248	0.957279	0.346473	0.085*	
C5	0.5901 (3)	0.89393 (15)	0.3833 (2)	0.0696 (6)	
H5	0.579890	0.938259	0.441639	0.083*	
C6	0.5065 (3)	0.81732 (14)	0.3555 (2)	0.0580 (5)	
H6	0.439261	0.809307	0.394462	0.070*	
C7	0.6186 (2)	0.52796 (12)	0.37144 (19)	0.0463 (4)	
C8	0.8002 (2)	0.49089 (16)	0.3045 (2)	0.0579 (5)	
C10	0.7564 (2)	0.40722 (14)	0.4752 (2)	0.0568 (5)	
H10	0.780068	0.359996	0.539203	0.068*	
C11	0.8767 (3)	0.5141 (2)	0.2123 (3)	0.0835 (8)	
H11A	0.853870	0.469409	0.140165	0.125*	
H11B	0.979663	0.514976	0.266457	0.125*	
H11C	0.845364	0.572710	0.171410	0.125*	
N1	0.50291 (19)	0.58192 (11)	0.35938 (18)	0.0508 (4)	
N2	0.69070 (18)	0.54638 (11)	0.29291 (17)	0.0537 (4)	
N3	0.64728 (17)	0.46152 (10)	0.46533 (16)	0.0496 (4)	
O1	0.28821 (16)	0.67414 (10)	0.25840 (16)	0.0653 (4)	
O2	0.39885 (17)	0.61859 (10)	0.10316 (14)	0.0621 (4)	
S1	0.41467 (5)	0.65419 (3)	0.23421 (5)	0.04929 (17)	
C9	0.8367 (2)	0.41647 (16)	0.3968 (2)	0.0600 (5)	0.591 (11)
C12	0.9420 (7)	0.3432 (4)	0.3990 (8)	0.0750 (19)	0.591 (11)
C13	0.9860 (17)	0.2756 (10)	0.5150 (18)	0.098 (4)	0.591 (11)
H13A	1.053800	0.233375	0.505298	0.148*	0.591 (11)
H13B	0.901665	0.243536	0.511702	0.148*	0.591 (11)
H13C	1.030623	0.306600	0.602434	0.148*	0.591 (11)
O3	0.9931 (8)	0.3397 (4)	0.3128 (8)	0.110 (2)	0.591 (11)
C9A	0.8367 (2)	0.41647 (16)	0.3968 (2)	0.0600 (5)	0.409 (11)
C12A	0.9727 (9)	0.3620 (7)	0.4306 (12)	0.079 (3)	0.409 (11)
C13A	0.990 (2)	0.2750 (12)	0.509 (3)	0.089 (4)	0.409 (11)
H13D	1.082486	0.248722	0.525215	0.133*	0.409 (11)
H13E	0.913988	0.233796	0.454891	0.133*	0.409 (11)
H13F	0.983594	0.286757	0.595831	0.133*	0.409 (11)
O3A	1.0616 (8)	0.3857 (7)	0.3876 (10)	0.106 (3)	0.409 (11)
H1	0.458 (2)	0.5700 (14)	0.409 (2)	0.054 (6)*	

### Atomic displacement parameters (Å^2^)

**Table:**

	*U*^11^	*U*^22^	*U*^33^	*U*^12^	*U*^13^	*U*^23^
C1	0.0557 (11)	0.0456 (9)	0.0403 (10)	0.0056 (8)	0.0228 (9)	0.0082 (7)
C2	0.0619 (13)	0.0559 (11)	0.0551 (12)	0.0062 (9)	0.0311 (10)	0.0070 (9)
C3	0.0567 (13)	0.0676 (14)	0.0760 (16)	0.0015 (10)	0.0320 (12)	0.0183 (11)
C4	0.0695 (15)	0.0511 (12)	0.0789 (17)	−0.0026 (10)	0.0157 (13)	0.0080 (11)
C5	0.0897 (18)	0.0535 (12)	0.0634 (14)	0.0051 (11)	0.0287 (13)	−0.0043 (10)
C6	0.0754 (15)	0.0552 (11)	0.0510 (12)	0.0089 (10)	0.0335 (11)	0.0040 (9)
C7	0.0469 (11)	0.0518 (10)	0.0433 (10)	0.0003 (8)	0.0215 (9)	0.0043 (7)
C8	0.0491 (12)	0.0804 (14)	0.0498 (12)	−0.0014 (10)	0.0258 (10)	0.0012 (10)
C10	0.0515 (12)	0.0632 (12)	0.0570 (12)	0.0109 (9)	0.0233 (10)	0.0133 (9)
C11	0.0691 (16)	0.125 (2)	0.0738 (17)	0.0090 (15)	0.0472 (14)	0.0168 (15)
N1	0.0595 (10)	0.0514 (9)	0.0521 (10)	0.0093 (7)	0.0336 (8)	0.0141 (7)
N2	0.0550 (10)	0.0635 (10)	0.0499 (10)	−0.0001 (8)	0.0289 (8)	0.0072 (7)
N3	0.0506 (9)	0.0558 (9)	0.0481 (9)	0.0081 (7)	0.0259 (8)	0.0112 (7)
O1	0.0542 (9)	0.0753 (9)	0.0733 (10)	0.0134 (7)	0.0331 (8)	0.0233 (8)
O2	0.0792 (11)	0.0595 (8)	0.0457 (8)	−0.0060 (7)	0.0234 (7)	0.0020 (6)
S1	0.0546 (3)	0.0506 (3)	0.0465 (3)	0.0042 (2)	0.0244 (2)	0.00978 (19)
C9	0.0466 (11)	0.0798 (14)	0.0577 (13)	0.0102 (10)	0.0254 (10)	0.0069 (10)
C12	0.045 (2)	0.101 (3)	0.084 (4)	0.011 (2)	0.030 (3)	0.006 (3)
C13	0.081 (6)	0.098 (5)	0.094 (6)	0.048 (5)	0.012 (5)	0.016 (5)
O3	0.096 (4)	0.128 (4)	0.144 (5)	0.037 (3)	0.087 (4)	0.017 (3)
C9A	0.0466 (11)	0.0798 (14)	0.0577 (13)	0.0102 (10)	0.0254 (10)	0.0069 (10)
C12A	0.054 (4)	0.105 (4)	0.077 (4)	0.025 (4)	0.025 (4)	0.001 (4)
C13A	0.072 (7)	0.107 (8)	0.086 (7)	0.025 (7)	0.032 (6)	−0.007 (7)
O3A	0.070 (4)	0.144 (6)	0.127 (6)	0.026 (4)	0.062 (4)	0.009 (4)

### Geometric parameters (Å, º)

**Table:**

C1—C2	1.378 (3)	C10—C9	1.376 (3)
C1—C6	1.382 (3)	C10—H10	0.9300
C1—S1	1.7538 (19)	C11—H11A	0.9600
C2—C3	1.388 (3)	C11—H11B	0.9600
C2—H2	0.9300	C11—H11C	0.9600
C3—C4	1.373 (3)	N1—S1	1.6465 (16)
C3—H3	0.9300	N1—H1	0.83 (2)
C4—C5	1.367 (3)	O1—S1	1.4267 (15)
C4—H4	0.9300	O2—S1	1.4226 (15)
C5—C6	1.368 (3)	C9—C12	1.507 (4)
C5—H5	0.9300	C12—O3	1.211 (5)
C6—H6	0.9300	C12—C13	1.497 (6)
C7—N2	1.330 (2)	C13—H13A	0.9600
C7—N3	1.337 (2)	C13—H13B	0.9600
C7—N1	1.374 (2)	C13—H13C	0.9600
C8—N2	1.338 (3)	C9A—C12A	1.501 (5)
C8—C9A	1.412 (3)	C12A—O3A	1.207 (5)
C8—C9	1.412 (3)	C12A—C13A	1.495 (6)
C8—C11	1.502 (3)	C13A—H13D	0.9600
C10—N3	1.329 (2)	C13A—H13E	0.9600
C10—C9A	1.376 (3)	C13A—H13F	0.9600
			
C2—C1—C6	121.33 (19)	C7—N1—S1	127.69 (14)
C2—C1—S1	120.01 (15)	C7—N1—H1	118.2 (14)
C6—C1—S1	118.66 (15)	S1—N1—H1	112.6 (15)
C1—C2—C3	118.4 (2)	C7—N2—C8	117.10 (17)
C1—C2—H2	120.8	C10—N3—C7	115.02 (17)
C3—C2—H2	120.8	O2—S1—O1	119.43 (10)
C4—C3—C2	120.1 (2)	O2—S1—N1	110.41 (9)
C4—C3—H3	120.0	O1—S1—N1	102.84 (9)
C2—C3—H3	120.0	O2—S1—C1	108.71 (9)
C5—C4—C3	120.8 (2)	O1—S1—C1	108.55 (9)
C5—C4—H4	119.6	N1—S1—C1	106.06 (9)
C3—C4—H4	119.6	C10—C9—C8	115.92 (19)
C4—C5—C6	120.2 (2)	C10—C9—C12	120.0 (3)
C4—C5—H5	119.9	C8—C9—C12	123.6 (3)
C6—C5—H5	119.9	O3—C12—C13	120.4 (5)
C5—C6—C1	119.3 (2)	O3—C12—C9	121.9 (4)
C5—C6—H6	120.3	C13—C12—C9	117.6 (5)
C1—C6—H6	120.3	C12—C13—H13A	109.5
N2—C7—N3	126.86 (17)	C12—C13—H13B	109.5
N2—C7—N1	118.71 (17)	H13A—C13—H13B	109.5
N3—C7—N1	114.43 (16)	C12—C13—H13C	109.5
N2—C8—C9A	120.86 (18)	H13A—C13—H13C	109.5
N2—C8—C9	120.86 (18)	H13B—C13—H13C	109.5
N2—C8—C11	114.9 (2)	C10—C9A—C8	115.92 (19)
C9A—C8—C11	124.2 (2)	C10—C9A—C12A	120.4 (3)
C9—C8—C11	124.2 (2)	C8—C9A—C12A	122.5 (3)
N3—C10—C9A	124.15 (19)	O3A—C12A—C13A	121.1 (5)
N3—C10—C9	124.15 (19)	O3A—C12A—C9A	120.1 (5)
N3—C10—H10	117.9	C13A—C12A—C9A	118.6 (6)
C9—C10—H10	117.9	C12A—C13A—H13D	109.5
C8—C11—H11A	109.5	C12A—C13A—H13E	109.5
C8—C11—H11B	109.5	H13D—C13A—H13E	109.5
H11A—C11—H11B	109.5	C12A—C13A—H13F	109.5
C8—C11—H11C	109.5	H13D—C13A—H13F	109.5
H11A—C11—H11C	109.5	H13E—C13A—H13F	109.5
H11B—C11—H11C	109.5		

### Hydrogen-bond geometry (Å, º)

**Table:**

*D*—H···*A*	*D*—H	H···*A*	*D*···*A*	*D*—H···*A*
N1—H1···N3^i^	0.83 (2)	2.06 (2)	2.891 (2)	179 (2)
C6—H6···O2^ii^	0.93	2.62	3.338 (3)	135
C10—H10···O1^i^	0.93	2.54	3.243 (3)	133
C11—H11*B*···O3*A*	0.96	2.26	2.769 (7)	113
C13*A*—H13*E*···O1^iii^	0.96	2.50	3.40 (3)	156

Symmetry codes: (i) −*x*+1, −*y*+1, −*z*+1; (ii) *x*, −*y*+3/2, *z*+1/2; (iii) −*x*+1, *y*−1/2, −*z*+1/2.
